# On the Shape of the Force-Velocity Relationship in Skeletal Muscles: The Linear, the Hyperbolic, and the Double-Hyperbolic

**DOI:** 10.3389/fphys.2019.00769

**Published:** 2019-06-19

**Authors:** Julian Alcazar, Robert Csapo, Ignacio Ara, Luis M. Alegre

**Affiliations:** ^1^GENUD Toledo Research Group, Universidad de Castilla-La Mancha, Toledo, Spain; ^2^CIBER of Frailty and Healthy Aging (CIBERFES), Madrid, Spain; ^3^Research Unit for Orthopaedic Sports Medicine and Injury Prevention, ISAG, University for Health Sciences, Medical Informatics and Technology, Hall, Austria

**Keywords:** torque-velocity, maximal unloaded shortening velocity, muscle power, Hill’s equation, Edman’s equation, muscle contraction, contraction velocity, motor unit

## Abstract

The shape of the force-velocity (F-V) relationship has important implications for different aspects of muscle physiology, such as muscle efficiency and fatigue, the understanding of the pathophysiology of several myopathies or the mechanisms of muscle contraction *per se*, and may be of relevance for other fields, such as the development of robotics and prosthetic applications featuring natural muscle-like properties. However, different opinions regarding the shape of the F-V relationship and the underlying mechanisms exist in the literature. In this review, we summarize relevant evidence on the shape of the F-V relationship obtained over the last century. Studies performed at multiple scales ranging from the sarcomere to the organism level have described the concentric F-V relationship as linear, hyperbolic or double-hyperbolic. While the F-V relationship has most frequently been described as a rectangular hyperbola, a large number of studies have found deviations from the hyperbolic function at both ends of the F-V relation. Indeed, current evidence suggests that the F-V relation in skeletal muscles follows a double-hyperbolic pattern, with a breakpoint located at very high forces/low velocities, which may be a direct consequence of the kinetic properties of myofilament cross-bridge formation. Deviations at low forces/high velocities, by contrast, may be related to a recently discovered, calcium-independent regulatory mechanism of muscle contraction, which may also explain the low metabolic cost of very fast muscle shortening contractions. Controversial results have also been reported regarding the eccentric F-V relationship, with studies in prepared muscle specimens suggesting that maximum eccentric force is substantially greater than isometric force, whereas *in vivo* studies in humans show only a modest increase, no change, or even a decrease in force in lengthening contractions. This review discusses possible reasons reported in the literature for these discrepant findings, including the testing procedures (familiarization, pre-load condition, and temperature) and a potential neural inhibition at higher lengthening velocities. Finally, some unresolved questions and recommendations for F-V testing in humans are reported at the end of this document.

## Introduction

The slower a skeletal muscle shortens the greater the force it can generate during contraction and *vice versa*. This force-velocity (F-V) relationship is a fundamental principle of skeletal muscle physiology that was derived based on Hill’s ground-breaking studies in isolated frog muscles (Hill, 1938) and originally used to develop theories of the mechanisms of skeletal muscle contraction (Huxley, 1957). In recent years, several studies have investigated the *in vivo* F-V relationship in an attempt to describe muscle function in complex tasks in elite sports (e.g., performance in vertical jumping, sprinting, or rowing) (Dorel et al., 2005; Cross et al., 2015; Jimenez-Reyes et al., 2016; Giroux et al., 2017) or activities of daily living in older adults (e.g., gait or chair rising ability) (Alcazar et al., 2018c). Moreover, there is an increased interest in applying the *in vivo* F-V relationship as a tool to guide training practice (Samozino et al., 2012; Morin and Samozino, 2016). The F-V relation can be evaluated by measuring the force produced by muscles at different active shortening or lengthening velocities, or the velocity at which muscles shorten or lengthen against different isotonic or auxotonic^1^ forces. Then, a mathematical function is usually fitted to the collected F-V points, from which several performance characteristics can be obtained either directly or through extrapolation, such as the maximal isometric force (*P*_0_), maximal unloaded shortening velocity (*V*_max_), and maximal power output (*W*_max_).

One criterion that has received comparatively little attention is the curvature of the F-V plot. While the F-V relationship is widely recognized as an important characteristic of muscle function, disagreement exists regarding its precise shape and the underlying mechanisms accounting for it. The F-V relationship has been suggested to be linear (Bobbert, 2012), hyperbolic (Hill, 1938), and double-hyperbolic (Edman, 1988a). These controversial opinions have important implications for F-V testing and the perception of contraction and adaptations of muscle function. The curvature of the F-V relationship is related to the maximal power output of skeletal muscles (Gordon et al., 1966), reflects the thermodynamic efficiency of contraction (Hill, 1964b; Woledge, 1968; Barclay, 2017) and may be used to study the consequences of muscle fatigue (Jones, 2010). The dependence of force generation from contraction velocity is so universally accepted that models explaining the mechanisms of skeletal muscle are specifically tested for their ability to predict the shape of experimentally determined F-V curves (Huxley, 1957; Piazzesi et al., 2007). Moreover, a better understanding of the F-V relationship and the underlying contraction mechanism might help elucidate the pathogenesis of several hereditary sarcomere myopathies and the effects of different drugs with effects on muscle contraction (Ferrantini et al., 2009; Malik et al., 2011; Mansson, 2014; Spudich, 2014), and ultimately benefit the development of robotic and prosthetic applications that rely on bio-inspired actuators that exhibit natural (muscle-like) features in order to mimic the performance of biological systems (Paluska and Herr, 2006; Schmitt et al., 2012; Xiong et al., 2014; Chen et al., 2016; Hyun et al., 2017).

In order to critically discuss the discrepant F-V relationships presented in the literature and the physiological mechanisms assumed to be responsible for their specific shapes, we reviewed the most relevant studies conducted on the F-V relationship of skeletal muscles during the last century. The following search terms and operators were used in the PubMed and Web of Science online databases: (“force” OR “torque”) AND (“velocity” OR “speed”). Additional suitable studies were included by screening the reference lists of the reviewed studies and other relevant reviews. A wide range of studies from *in vitro* motility assays to *in vivo* human studies are presented in mostly chronological order.

## First Studies on the F-V Relationship

**FIGURE 1:**
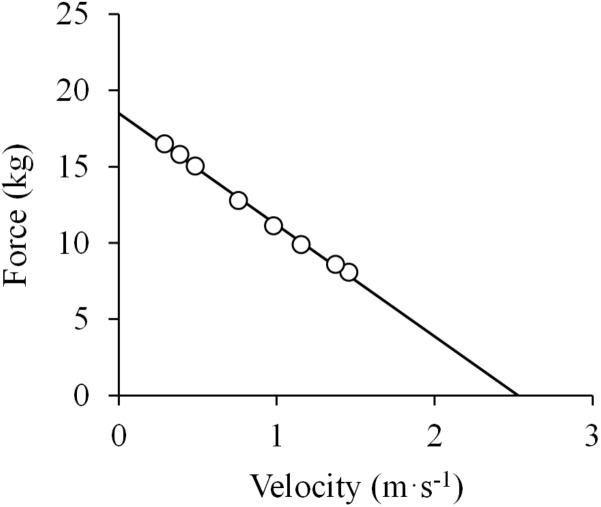
Linear force-velocity relationship. Data were obtained from Figure 4 in Hill (1922) using specialized software (ImageJ 1.51q8, NIH, United States). This modified version represents the force-velocity relationship of a standard subject during elbow flexions. From those data, force was calculated as the ratio between mechanical work and the range of movement (ROM) during the exercise (0.6 m), and velocity as the ROM divided by the time registered for each repetition. A linear function was fitted to the data (least squares method): *F* = –7.3 ×*V* + 18.5; *R*^2^ = 0.997.

The earliest reference to the F-V relationship is perhaps that made by Hill (1922): “the force exerted is greater the less the rate of movement, and *vice versa.*” In that study, Hill presented a novel instrument created to evaluate the *in vivo* mechanical work of human muscles. A hand tachometer measured the revolution speed of a fly-wheel pulled by means of a handle attached to an inelastic string, and the kinetic energy of the fly-wheel was obtained. The resistance could be modified by winding the string around different sized pulleys of the fly-wheel. In the experiment, the participants performed several concentric elbow flexions as powerfully as possible against different resistances, which is an important criterion when evaluating the F-V relationship due to its implications for motor unit recruitment and maximal force production (discussed later). The range of movement (ROM) was controlled by adjusting the length of the string to each participant and kept constant for all repetitions. From the mechanical work and time data reported in that study (Hill, 1922), a mechanical work-velocity relationship can be obtained, and since the ROM was constant between repetitions, the F-V relationship can also be elucidated. An image processing and analysis software (ImageJ 1.51j8, NIH, United States) was used to extract the data presented by Hill in Figure 4 of that study (Hill, 1922). Those data showed that the F-V relationship obtained from average force and velocity values could be described as a linear function (*R*^2^ = 0.997) with a negative slope (Figure 1). These findings were replicated by other studies of that time (Lupton, 1922; Hansen and Lindhard, 1923; Hill et al., 1924), which led Hill to suppose that the mechanical behavior of muscle was determined by the viscosity of its contractile material (Hill, 1950). According to this rationale, higher shortening velocities would require the muscle to overcome higher resistances, thus lowering the force it could produce. However, acknowledging that the observed linearity of the F-V relation could also be determined by neural mechanisms, Hill expanded his studies.

Gasser and Hill (1924) carried out a series of experiments in isolated frog muscles in order to ascertain the relations established in earlier studies (Hill, 1922; Lupton, 1922; Hansen and Lindhard, 1923; Hill et al., 1924). In these experiments the authors used a previously described apparatus (Doi, 1921) by which muscles were attached to one end of a first class lever and then electrically stimulated to eliminate potential influences of the central nervous system. The authors found that “as in the experiments on man, the work (force) performed decreases as the speed increases,” but “in the isolated muscle the relation between work (force) and speed is not linear, and differs in this respect from that in human arm muscles” (Gasser and Hill, 1924). However, work was calculated as the product of weight and distance (rather than as the product of force and distance), thus considering the potential energy but neglecting the kinetic energy of the lever just before hitting one of the stops of the apparatus. Consequently, division by distance would not result in an F-V but rather a load-velocity relationship. Nevertheless, additional experiments carried out with the tension exerted by the muscle being measured by an isometric tension recorder confirmed that at increasing velocities the capacity of muscles to produce force declines in a curvilinear rather than in a linear fashion (Gasser and Hill, 1924). A curvilinear relation was also noted by Levin and Wyman (1927), whose pioneering work also provided information on the tension produced by different sorts of animal muscles at various stretching velocities (i.e., in eccentric contractions), which led the authors to conclude that the resulting curve was S-shaped, with eccentric force being greater than concentric force. In the light of this new evidence showing a curvilinear F-V relationship in isolated muscle specimens, the viscosity theory was abandoned and replaced by a viscoelastic theory to explain the mechanical behavior of skeletal muscle (Hill, 1950).

Fenn and Marsh (1935) were the first to conduct a series of experiments focusing on the F-V relationship of isolated animal muscles, as opposed to all previous studies determining the relationship between mechanical work and velocity. Their experiments consisted of measurements of the maximum velocity of shortening with a series of different loads. The measurements were always made at the same muscle length, in a region where velocity was found to be maximal and nearly constant. Since there was no change in velocity at that point, the force exerted by the muscle must be equal to the load. With respect to the shape of the F-V relationship, the authors concluded that “it is obvious that no portion of this curve is linear but it is rather logarithmic in shape” (Fenn and Marsh, 1935). Importantly, based on the observation of the temperature-dependency of their results, Fenn and Marsh suggested that some chemical reactions must have an effect on the degree of force loss that occurs with increasing velocity, and thus, that the muscle could not properly be considered a simple mechanical (i.e., viscoelastic) system, as previously assumed.

## The Hyperbolic Force-Velocity Relationship

**FIGURE 2:**
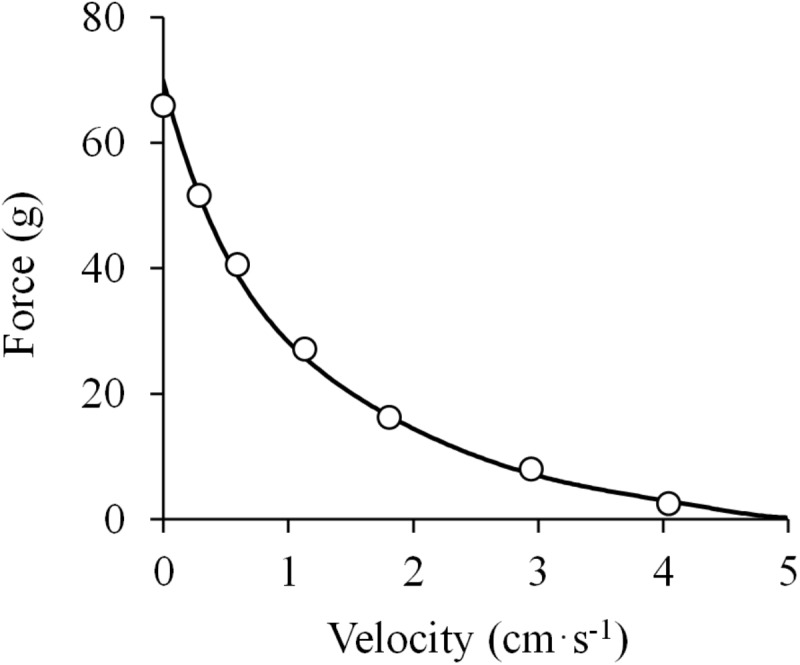
Hyperbolic force-velocity relationship. Data were obtained from Figure 12 in Hill (1938) using specialized software (ImageJ 1.51q8, NIH, United States). This modified version represents the force-velocity relationship of the isolated sartorius muscle of a frog. A hyperbolic function was fitted to the data (least squares method): (*F* + 14.4) × (*V* + 1.0) = (70.6 + 14.4) × 1.0; *R*^2^ = 0.998.

Hill (1938) published his seminal work “The heat of shortening and the dynamic constants of muscle.” The author utilized a more accurate and rapid technique for the evaluation of the effect of load on the shortening velocity of muscle by means of a device combining a thermopile, a galvanometer and an oscilloscope. When a muscle was tetanized isometrically, a continuous heat record was obtained that was considered as the baseline signal. When the muscle was allowed to shorten, the heat of contraction rose above the level observed during the isometric contraction. The increase in temperature was considered to be proportional to the degree of shortening, and thus velocity was recorded continuously during isotonic contractions. Since the apparatus used a system of mounted levers, the kinetic energy was considered small enough to be neglected, and load was assumed to be equivalent to the force produced by the muscle. Therefore, the relation between load (force) and velocity in isotonic concentric muscle actions was measured, which showed a curvilinear pattern better represented by a rectangular hyperbola (Figure 2). This hyperbola is given by the equation (Hill, 1938):

(1) $$(P+a)(V+b)=(P_{0}+a)b$$

where *P* is load, *V* is velocity, *P_0_* is the isometric force, *a* is a constant indicating the shortening heat per unit of shortening with the dimensions of load, and *b* is a constant expressing the increase of energy rate per unit of decrease in load with the dimensions of velocity. The ratio *a*/*P_0_* indicates the curvature of the F-V relationship, which Hill believed to represent a fundamental constant of muscle in humans and other species alike (Hill, 1940). Importantly, this experimental model was the first to consider both the material properties of muscle as well as possible chemical reactions (estimated by heat measurements) influencing the process of muscle contraction. This equation was also reported to fit satisfactorily with the previous experiments conducted by Fenn and Marsh (1935).

During the following decades, more studies were conducted in order to confirm the results reported by Hill (1938). Katz (1939) conducted a series of experiments in which the shortening and lengthening velocity of muscle was evaluated at forces both lower and higher than *P*_0_. The concentric portion of the F-V relationship (positive velocities) was reported to be adequately fitted by Hill’s hyperbolic equation. While Hill’s F-V relation was generally accepted as a model of the concentric F-V relationship of skeletal muscle, Katz complemented Hill’s theory by making some remarkable observations. Katz demonstrated that changes in temperature affected the curvature of the F-V relationship by diminishing the value *a*/*P_0_* with increasing temperature (Katz, 1939). He also found that *a*/*P*_0_ could vary between species, thus refuting Hill’s notion of it being a species-independent constant of muscle (Katz, 1939). Later studies confirmed that *a*/*P*_0_ varies with different muscle lengths, temperatures and excitation levels, and also between different species and individuals of the same species (Ralston et al., 1949; Wilkie, 1949; Abbott and Wilkie, 1953; Bigland and Lippold, 1954). Moreover, Katz noted that velocities at forces greater than the isometric force were considerably smaller than those predicted by Hill’s model, which could therefore not be applied to the eccentric portion of the F-V relationship (Katz, 1939). Of note, heat changes during lengthening contractions were found to be too small to be accurately captured (Hill, 1960). In line with this observation, decreased muscle excitation levels were observed during active muscle lengthening (Bigland and Lippold, 1954), demonstrating physiological differences between eccentric and concentric muscle actions.

**FIGURE 3:**
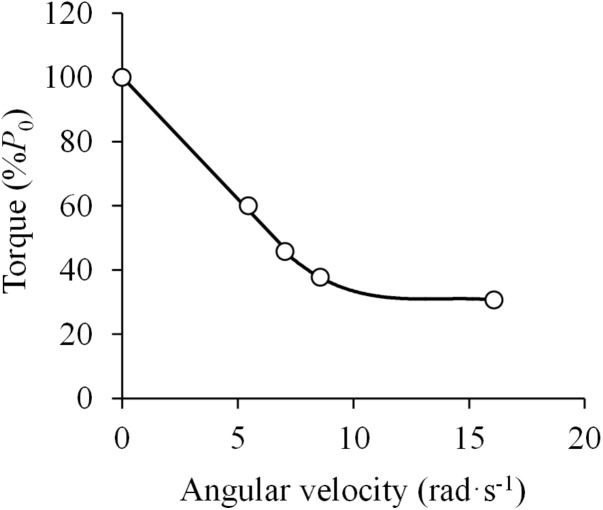
Force (torque)-velocity (angular velocity) relationship. Data were obtained from Figure 8 in Dern et al. (1947) using specialized software (ImageJ 1.51q8, NIH, United States). This modified version represents the force-velocity relationship of a standard subject during elbow flexions. *P*_0_ denotes the maximal isometric torque. The attempts with no apparent effect of fatigue were selected. A linear function was fitted to the data above 40% of *P*_0_ (*F* = –7.6 ×*V* + 100.3; *R*^2^ = 0.998) and a third order polynomial function was fitted to the data below 40% of *P*_0_ (*F* = –0.05 ×*V*^3^ + 2.00 ×*V*^2^ – 27.67 ×*V* + 158.10; *R*^2^ = 1.000) (least squares method in both).

The first study that aimed to verify the applicability of Hill’s equation to *in vivo* human muscle was that carried out by Dern et al. (1947), who tested the F-V relationship of the elbow flexor muscles by having subjects perform maximally explosive contractions against varying resistance. The authors reported that the F-V relationship was best represented by a curvilinear function, but these results were affected by apparent effects of fatigue. Had only the best attempts (i.e., the trials not affected by fatigue) been included into the analysis, the F-V relation would instead be nearly linear at torque values greater than 40%, and display a curvilinear pattern below that level (Dern et al., 1947) (Figure 3).

**FIGURE 4:**
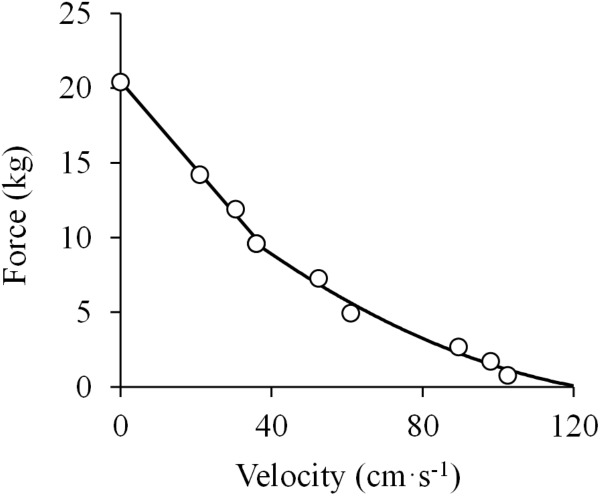
Force-velocity relationship. Data were obtained from Figure 1 in Ralston et al. (1949) using specialized software (ImageJ 1.51q8, NIH, United States). This modified version represents the force-velocity relationship of the *in vivo* human pectoralis major muscle. A linear function was fitted to the data above 40% of maximal isometric force (*P*_0_) (*F* = –0.29 × *V* + 20.43; *R*^2^ = 0.996) and a second order polynomial function was fitted to the data below 40% of *P*_0_ (*F* = 0.0008 ×*V*^2^ – 0.2335 ×*V* + 16.991; *R*^2^ = 0.983) (least squares method in both).

The next work to address the F-V relationship was a unique study performed with amputee men with cineplastic^2^ tunnels through various muscles of the upper extremity, making it possible to evaluate the *in vivo* F-V relationship of isolated, yet voluntarily contracting human muscles (Ralston et al., 1949). The authors evaluated the peak velocity attained by the pectoralis major muscle contracting against different loads, and the resultant F-V relationship was reported to fit well with Hill’s rectangular hyperbola. However, *post hoc* analyses of the respective data performed by us showed that the F-V data by Ralston et al. (1949) are best represented by a linear function at forces higher than 40% of the isometric peak force (*R*^2^ = 0.996), while they follow a curvilinear function below that level of force (*R*^2^ = 0.986) (Figure 4). These analyses negate a purely hyperbolic F-V relation.

By contrast, Wilkie (1949) reported a Hill-type load-velocity relationship for the human elbow flexor muscles. In our review of this study, we did not observe a clear linear pattern in the region of the F-V relationship that comprised forces between ∼40–100% of *P*_0_ as the measured *P*_0_ was higher than that expected from a linear fit. However, when the isometric force was not considered, the linear fitting was excellent (*R*^2^ = 0.998) for the three loads higher than ∼50% of the isometric force.

**FIGURE 5:**
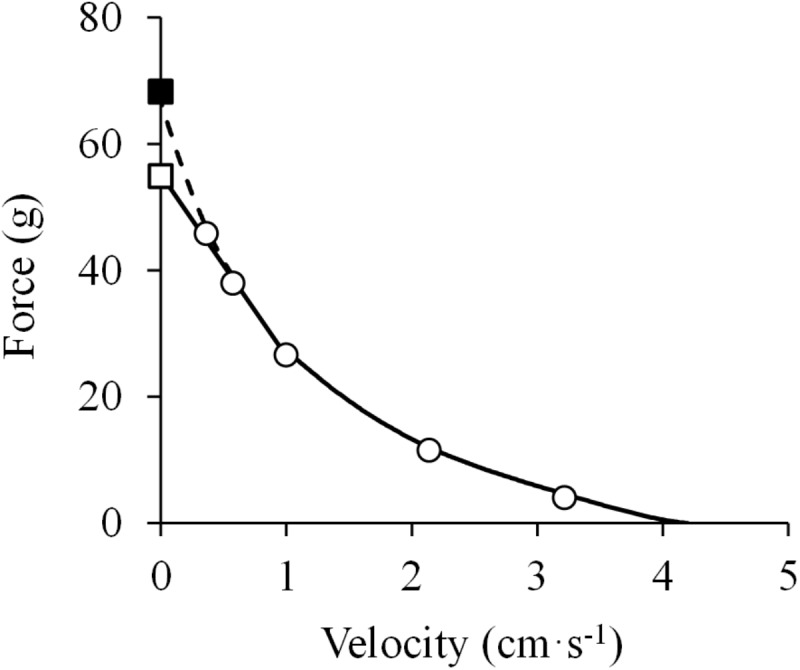
Force-velocity relationship. Data were obtained from Figure 5 in Abbott and Wilkie (1953) using specialized software (ImageJ 1.51q8, NIH, United States). This modified version represents the force-velocity relationship of the isolated sartorius muscle of a frog. When the baseline isometric force was considered (*closed square*), a hyperbolic equation was fitted to the data [(*F* + 18.5) × (*V* + 1.2) = (67.0 + 18.5) × 1.2; *R*^2^ = 0.999] (*dashed line* in the high-force region) (least squares method). In contrast, when the isometric force measured after the isotonic recordings was considered (*open square*), a linear model was adequately fitted to the data above 40% of maximal isometric force (*P*_0_) (*F* = –28.8 ×*V* + 55.3; *R*^2^ = 0.996) (*solid line* in the high-force region), while the hyperbolic function was adequately fitted to the F-V data below 40% of *P*_0_ (*R*^2^ = 0.999) (*solid line* in the low-force region) (least squares method in both).

Jointly, it appears that studies reporting a linear F-V relationship had only measured forces greater than 40% (Hill, 1922; Lupton, 1922; Hansen and Lindhard, 1923; Hill et al., 1924), while others concluding that the F-V relation was hyperbolic assessed forces both lower and higher than 40% of the isometric force (Gasser and Hill, 1924; Levin and Wyman, 1927; Fenn and Marsh, 1935; Hill, 1938; Katz, 1939; Dern et al., 1947; Ralston et al., 1949; Wilkie, 1949). Consequently, the F-V relationship could follow a pattern that is linear at intermediate to high forces (∼40–100%) but deviates upwards at low forces (∼0–40%) to become curvilinear. While the linearity of the F-V relationship at high forces was not clearly evident in the studies conducted by Hill (1938) and Wilkie (1949), an explanation might be provided by a later study by Abbott and Wilkie (1953). In their experiments, after completion of the dynamic contractions, the authors noted not only a decrease in maximal isometric force but also a shift of the optimal length to longer muscle lengths (Abbott and Wilkie, 1953). A similar observation was reported by Ritchie (1954), who recorded isometric force in isolated rat muscle during every third or fourth isotonic contraction, and found that isometric force decreased toward the end of the series of tests performed with increasing loads. In order to accord with Hill’s theory, the authors deliberately selected those isometric force values that gave the best fit for a hyperbolic F-V relation (Abbott and Wilkie, 1953; Ritchie, 1954), while a clear linear pattern (*R*^2^ = 0.996, at forces > 40% of *P*_0_) may be observed when other isometric values obtained during the experiments are chosen (Figure 5). These biased conclusions may have hindered the correct analysis of the high-force/low-velocity region of the F-V relationship. In some previous studies, the isometric force was not evaluated (Gasser and Hill, 1924; Levin and Wyman, 1927; Fenn and Marsh, 1935) or it was only measured at the beginning of the experiments (Hill, 1938; Katz, 1939). In addition, in experiments performed in isolated muscles, the isometric force was measured at the optimal length, while velocity values during dynamic actions were registered after the muscle had shortened a certain distance (Hill, 1938; Katz, 1939). Thus, isometric force and dynamic F-V data were collected at slightly different muscle lengths, leading to an overestimation of *P*_0_. Consequently, Ritchie and Wilkie (1958) proposed that previous studies might have missed deviations from the rectangular hyperbola because of the considerable uncertainty of the appropriate value of the isometric tension.

The success of Hill’s equation (Hill, 1938) over other mathematical models similarly fitting reasonably well with empirical F-V data (Fenn and Marsh, 1935; Polissar, 1952; Abbott and Wilkie, 1953; Aubert, 1956) was due to its ability to integrate mechanical and thermal parameters. Hill’s theory was so well-accepted that many authors were keen to demonstrate the congruence of their F-V data with the original hyperbolic F-V relationship (Hill, 1938), perhaps in an attempt to demonstrate the correctness of their results. However, some of Hill’s assumptions were found to be incorrect. For example, *a*/*P*_0_ (i.e., the curvature of the F-V relationship) was demonstrated not to be a fundamental constant of muscle, rather it could vary depending on the species, temperature (*a*/*P*_0_ decreases with increasing temperature) (Katz, 1939), type of muscle (Close, 1964), excitation level (*a*/*P*_0_ decreases with increasing muscle excitation) (Bigland and Lippold, 1954) and muscle length (*a*/*P*_0_ decreases as muscle length approximates to the optimal length) (Abbott and Wilkie, 1953), and also between participants (Wilkie, 1949). The constant *a* of Hill’s equation was found to represent the degree of muscle shortening, but not the heat of shortening obtained from thermal measurements (Aubert, 1952, 1956; Abbott and Lowy, 1956; Hill, 1964a). In addition, despite the generally satisfactory fitting of the hyperbolic F-V function with experimental F-V data obtained below a certain level of force, evidence from studies conducted either in isolated muscle (Katz, 1939; Ralston et al., 1949; Abbott and Wilkie, 1953; Ritchie, 1954; Ritchie and Wilkie, 1958; Sonnenblick, 1962) or using *in situ* measurements (Dern et al., 1947; Allen and Stainsby, 1973; Komi, 1973; Thorstensson et al., 1976; Lannergren, 1978; Perrine and Edgerton, 1978; Wickiewicz et al., 1984; Froese and Houston, 1985) increasingly challenged the rectangular hyperbolic shape at high forces/low velocities. Even Hill himself pointed out that the F-V data might deviate from a hyperbola in the high-force region of the F-V relationship (Hill, 1970, pp 112–117). The main limitation of studies performed at that time was that none had specifically investigated the observed deviations from the rectangular hyperbola. In addition, few studies had described the eccentric portion of the F-V relationship (Levin and Wyman, 1927; Katz, 1939). Indeed, most studies did not evaluate the F-V relationship in the high-force range (i.e., forces > 75–80% of *P*_0_), and in some of them the maximal isometric force was not even measured.

## The Double-Hyperbolic Force-Velocity Relationship

**FIGURE 6:**
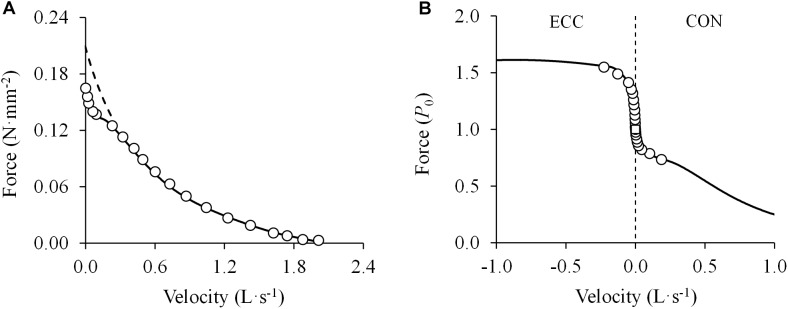
**(A)** Double-hyperbolic force-velocity relationship. Data were obtained from Figure 2 in Edman (1988a) using specialized software (ImageJ 1.51q8, NIH, United States). This modified version represents the force-velocity relationship of a single muscle fiber from the anterior tibialis muscle of a frog. Note the deviation of the experimental data from those predicted by the rectangular hyperbola in the high-force region (>0.78 maximal isometric force or *P*_0_) despite the excellent fit ((*F* + 0.06) × (*V* + 0.59) = (0.21 + 0.06) × 0.59; *R*^2^ = 0.994) (*dashed line*) (least squares method). In contrast, all measurement data are well-represented by a double-hyperbolic F-V equation ($V=\frac{(0.21-F)\times 0.59}{F+0.06}-(1-\frac{1}{1+e^{-154.65\times (F-0.82\times 0.17)}})$; *R*^2^ = 0.999) (*solid line*) (least squares method). **(B)** Sigmoidal transition of the force-velocity relationship from concentric (CON) to eccentric (ECC) dynamic muscle actions (*open circles*). Data were obtained from Figure 7 in Edman (1988a) using specialized software (ImageJ 1.51q8, NIH, United States). This modified version represents the eccentric and concentric force-velocity relationship of a single muscle fiber from the anterior tibialis muscle of a frog. A double-hyperbolic function was fitted to the concentric data (*see above*) and a hyperbolic function was fitted to the eccentric data ($V=\frac{0.028\times (1.000-F)}{2\times 1.000-F+(-0.384)}$; *R*^2^ = 0.990) (least squares method). Note the drastic differences in force around the isometric force (*open square*) (0.90–1.20 *P*_0_) with only minimal changes in contraction velocity (1.8% of maximal unloaded shortening velocity).

In the light of the previously mentioned limitations of Hill’s theory, Edman et al. (1976) conducted a study in 1976 to determine the shape of the F-V relationship and the possible factors that may account for deviations from a rectangular hyperbola reported in previous studies. The experiments were performed in single isolated frog muscle fibers and also in bundles of frog muscle fibers, and a sufficient number of experimental data points over the whole F-V relationship was obtained. The authors found that the F-V relationship systematically deviated from the rectangular hyperbola at forces above 78% of the measured isometric force (i.e., *P*_0_). Importantly, the deviation from the hyperbolic F-V relationship was found to be independent from the mode of activation or the time interval between tetani (1, 3, or 60 min) (Edman et al., 1976). Thus, the deviation from the hyperbola was found not to be caused by muscle fatigue. Interestingly, in spite of the evident and systematic deviation in the high-force/low-velocity region, the fitting of all F-V points by a rectangular hyperbola still showed a very high correlation coefficient (*r* = 0.9987). However, when the F-V data were truncated at 78% of *P*_0_ (i.e., the range of data that did not deviate from the rectangular hyperbola), the estimated isometric force ($P_{0}^{*}$) values were 32% higher than the experimentally determined ones, and average *a*/$P_{0}^{*}$ was 0.18 compared to 0.28 before truncation (Edman et al., 1976). The deviation of Hill’s hyperbola from empirical data at high forces was also confirmed in other studies (Bahler et al., 1968; Edman and Hwang, 1977; Edman, 1979; Edman et al., 1985). Further studies by Edman performed in single isolated frog muscle fibers and short segments along intact fibers (Edman, 1988a,b) finally showed that the F-V relation exhibited two distinct curvatures located on either side of a breakpoint near 78% of *P*_0_ and 11% of *V*_max_. F-V data truncated at 78% of *P*_0_ were excellently fitted by Hill’s hyperbolic function, but its derived isometric force (i.e., $P_{0}^{*}$) was substantially higher (+17%) than the measured isometric force (i.e., *P*_0_). The data at forces > 78% *P*_0_ could best be fitted with an independent hyperbola, and both hyperbolas were found to be closely related to each other (Figure 6A). Therefore, the F-V relationship was found to be better characterized by a double-hyperbolic F-V equation (Edman, 1988a):

(2) $$V=\frac{(P_{0}^{*}-P)b}{P+a}\left(1-\frac{1}{1+e^{-k_{1}(P-k_{2}P_{0})}}\right)$$

where the first term expresses the F-V relationship at low and intermediate forces (<0.78 *P*_0_) and $P_{0}^{*}$ is the isometric force that is predicted from the rectangular hyperbola derived from values below 0.78 *P*_0_; and the second term modifies the F-V relationship at high forces (>0.78 *P*_0_) with *k_1_* and *k_2_* as constants, determining the degree of curvature and the point of transition, respectively. The first term basically corresponds to Hill’s original equation, while the second term is a ‘correction term’ that reduces *V* in the high-force region of the F-V relation (Edman, 1988a). Importantly, the fact that the double-hyperbolic F-V relationship was observed in both the fiber as a whole and a short segment of the same fiber suggests that this pattern represented the contractile behavior at the sarcomere level. In addition, the F-V relationship was also investigated at forces that exceeded the isometric force (1.0–1.8 *P*_0_) (Edman, 1988a). The F-V relation formed a smooth sigmoidal function with inflection at *P*_0_, and was observed to be nearly flat between 0.9 and 1.2 *P*_0_ with velocity values differing by only 1.8% within this force range (Figure 6B). By contrast, changes in velocity were progressively greater at forces between 1.2 and 1.6 *P*_0_. Unlike the hyperbolic F-V equation (Katz, 1939), Edman’s F-V equation appeared to fit reasonably with the subsequent eccentric portion of the F-V relation (Edman, 1988a), although no equation was specifically created for this section of the F-V relationship. The curvatures of the two connected hyperbolas were found to decrease (but not disappear) at longer sarcomere lengths and with increases in temperature (Edman, 1988a), and they were consistently observed at the same relative values of *P*_0_ (80%) and *V*_max_ (10%) after depressing the isometric force to 80% of the control value by dantrolene (a substance known to reduce the release of calcium from the sarcoplasmic reticulum) (Edman, 1993).

The double-hyperbolic shape of the F-V relationship was confirmed in intact frog single muscle fibers (Iwamoto et al., 1990), skinned frog single muscle fibers (Lou and Sun, 1993), intact frog muscle spindles (Edman et al., 2002), intact mammalian single muscle fibers (Edman, 2005; Colombini et al., 2009) and fiber bundles (Roots et al., 2007), isolated mammalian skeletal muscles (Mansson et al., 1989), proximal and distal parts of mammalian skeletal muscles *in situ* (Rijkelijkhuizen et al., 2003), and even in smooth muscles (Wang et al., 1994). The first study evaluating whether the double-hyperbolic F-V relation might also be found in whole mammalian skeletal muscle *in situ* was that conducted by Devrome and MacIntosh (2007). A rat’s medial gastrocnemius muscle was surgically isolated while keeping the innervation, blood supply, temperature, and muscle origin intact. The sciatic nerve was electrically stimulated to elicit maximal muscle contractions against different loads ranging from nearly unloaded to maximal isometric force. The F-V relationship was found to display a double-hyperbolic shape, with a breakpoint located at 88% of *P*_0_ and 7.3% of *V*_max_ (Devrome and MacIntosh, 2007). After a fatiguing protocol, both the *P*_0_ and *V*_max_ values were significantly decreased, though the double-hyperbolic nature of the F-V relation was maintained. With fatigue the breakpoint was located at the same relative value of *P*_0_, but increased to 8.3% of *V*_max_ (Devrome and MacIntosh, 2007). These findings have recently been confirmed by the same authors, with the breakpoint of the double-hyperbolic F-V relationship located at 90% of *P*_0_, but this time it was decreased to 80% of *P*_0_ after a fatiguing protocol (Devrome and MacIntosh, 2018).

Importantly, the evidence demonstrating the double-hyperbolic shape of the F-V relationship lends support to the vast number of studies presented in this review that show a linear F-V relationship at forces greater than 40% of *P*_0_. When force values in the high-force/low-velocity region are substantially lower than those predicted by a rectangular hyperbola, the F-V relation may give the false impression of being linear between 40 and 100% of *P*_0_.

## Deviations From Hill’s Rectangular Hyperbola at Very Low Forces

The previous sections focused on the deviations from the rectangular hyperbola observed in the high-force/low-velocity region of the F-V relation. Nonetheless, deviations from the rectangular hyperbola have also been found at very high contraction velocities (Edman, 1979; Claflin and Faulkner, 1985). Maximum shortening velocity or the velocity of unloaded shortening (i.e., at zero load) is usually extrapolated from the F-V data (i.e., *V*_max_) assuming a hyperbolic relation (Hill, 1938). However, a more direct method to determine it is the ‘slack test,’ by which the maximum shortening velocity against zero load (i.e., *V*_0_) can be measured in isolated muscles or muscle fibers (Edman, 1979), but also during *in vivo* measurements conducted in humans (Sasaki and Ishii, 2005; Sasaki and Ishii, 2010). *V*_0_ is an important measure in that it reflects the kinetic properties of actomyosin interactions (Schiaffino and Reggiani, 1996; Bottinelli and Reggiani, 2000). However, *V*_0_ has been found to be greater than *V*_max_ in whole muscles (Edman, 1979; Claflin and Faulkner, 1985) due to the fact that *V*_0_ is a measure of the maximal unloaded shortening velocity of the fastest muscle fibers, whereas *V*_max_ is a function of the F-V relationship of all muscle fibers, provided that it is estimated from F-V data obtained at moderate loads. Thus, *V*_max_ and *V*_0_ are similar when measurement data are obtained at sufficiently low forces (Edman, 1979), while substantial differences may be found when *V*_max_ is obtained from F-V points relatively far from *V*_0_ (Claflin and Faulkner, 1985). Furthermore, high fiber type heterogeneity may augment the differences between *V*_max_ and *V*_0_ values because the presence of slower fibers would influence *V*_max_ estimations while not having any effect on *V*_0_ values (Josephson and Edman, 1988). This phenomenon may be explained by the differences in contraction velocity of slow- and fast-twitch fibers: since slower fibers cannot keep up with the contraction rate of their fast-twitch neighbors, they would not be able to contribute to the total muscle’s force production at high contraction velocities. Alternatively, one could speculate that the presence of slower muscle fibers might reduce the contraction velocity of the whole muscle since fibers are mechanically connected through the muscle’s extracellular matrix, thus facilitating a more uniform shortening of the entire muscle (Claflin and Faulkner, 1989). For this reason, the maximal unloaded shortening velocity of the whole muscle might be slightly lower (∼15%) than the maximal unloaded shortening velocity of its fastest motor units (Claflin and Faulkner, 1989), although slow fibers can shorten above their *V*_0_ values through the assisting forces provided by fast fibers simultaneously contracting in their vicinity (Edman, 2014). In any case, from the underestimation of the true maximal shortening velocity it follows that the F-V relationship is not strictly hyperbolic in the low-force region either (<0.05 *P*_0_) (Close and Luff, 1974; Julian et al., 1986; Josephson and Edman, 1988), which was also acknowledged by Hill (1970, pp 29–31).

## Studies on the *In Vivo* F-V Relationship in Humans

It is important to note that, in contrast to *in vitro* studies of isolated single muscle fibers or whole muscles, several factors other than cross-bridge kinetics influence the observed F-V relationship under *in vivo* conditions. These factors include neural activation, the mechanical properties of in-series elastic components, lateral force transmission between neighboring muscle fibers, muscle architecture, lever arms of joints, coordination of agonist and antagonist muscles and other possible factors that might be outside of our current understanding on muscle contraction and function. For example, it has been demonstrated that muscle moment arm length influences the torque-velocity relationship (Nagano and Komura, 2003). A longer moment arm requires muscle shortening velocity to be greater at any given joint angular velocity, thus forcing the muscle to act in a lower region of its F-V relationship. This detrimental effect on muscle force is compensated by the longer moment arm during slow joint angular velocities, and consequently greater joint moments were observed at slow joint angular velocities compared with having a shorter moment arm. In contrast, the decreased muscle force could not be compensated by the longer moment arm during fast joint angular velocities, resulting in lower joint moments (Nagano and Komura, 2003). These considerations notwithstanding, the evaluation of the *in vivo* F-V relationship is still of great relevance for muscle and exercise physiology, as the F-V curve reflects human performance (Dorel et al., 2005; Cross et al., 2015; Jimenez-Reyes et al., 2016; Giroux et al., 2017; Alcazar et al., 2018c) and may be used to guide training practice (Samozino et al., 2012; Morin and Samozino, 2016).

Deviations from the rectangular hyperbola are not unusual in the *in vivo* F-V relationship in humans during either single- (Komi, 1973; Thorstensson et al., 1976; Perrine and Edgerton, 1978; Wickiewicz et al., 1984; Froese and Houston, 1985; Dudley et al., 1990; Finni et al., 2003; Chino et al., 2008; Fontana Hde et al., 2014) or multi-joint muscle actions (Sargeant et al., 1981; Vandewalle et al., 1987; Beelen and Sargeant, 1991; Bosco et al., 1995; Rahmani et al., 2001, 2018; Hintzy et al., 2003; Macaluso and De Vito, 2003; Pearson et al., 2004; Yamauchi et al., 2007, 2009, 2010; Rejc et al., 2010; Bobbert, 2012; Samozino et al., 2012, 2014a,b; Cuk et al., 2014, 2016; Zbinden-Foncea et al., 2014; Jaric, 2015; Bobbert et al., 2016; Garcia-Ramos et al., 2016, 2018; Giroux et al., 2016; Alcazar et al., 2017, 2018c; Avrillon et al., 2017; Banyard et al., 2017; Fernandes et al., 2017; Padulo et al., 2017; Riviere et al., 2017; Zivkovic et al., 2017a,b; Navarro-Cruz et al., 2019). In both cases, the reason for the deviations was speculated to be a central inhibitory mechanism (Perrine and Edgerton, 1978; Wickiewicz et al., 1984; Yamauchi et al., 2007). This hypothesis was tested by several studies evaluating the *in vivo* human F-V relationship during isokinetic knee extensions elicited by maximal voluntary muscle actions versus neuromuscular electrical stimulation or superimposed electrical stimulation. No alterations in voluntary activation were observed during isometric or concentric knee extensions (Dudley et al., 1990; Westing et al., 1990, 1991; Pain and Forrester, 2009). These observations conflict with the hypothesis of neural inhibition being the factor explaining the deviation of force values from the hyperbolic F-V relationship at low concentric velocities. In other studies, the F-V relationship of plantar flexor and knee extensor muscles were adequately fitted by Hill’s hyperbolic equation (Hauraix et al., 2013, 2015, 2017). Nevertheless, joint angular velocities corresponding with the high-force/low-velocity region of the F-V relationship (below ∼30°⋅s^-1^) were not evaluated, and thus the existence of a double-hyperbolic F-V relationship was not assessed.

Interestingly, two studies focused specifically on the high-force/low-velocity region of the F-V relationship in the human knee extensor muscles (Harris and Dudley, 1994; Seger and Thorstensson, 2000). The F-V data presented by Harris and Dudley (1994), from both voluntary and electrically stimulated muscle actions, might correspond well with a double-hyperbolic F-V relationship. An upward-concave curvature in the high-force/low-velocity region of the F-V relationship was more evident at more extended compared to more flexed knee joint positions (i.e., at shorter muscle lengths) (Harris and Dudley, 1994), a feature previously also observed by Edman in isolated single muscle fibers (Edman, 1988a). In the study of Seger and Thorstensson (2000), the authors registered concentric and eccentric torque values at very low angular velocities (0, 10, 20, and 30°-⋅s^-1^). Their findings from both voluntary and electrically evoked muscle actions agreed with Edman’s observation of a sigmoidal transition zone between the concentric and eccentric part of the F-V relationship (Edman, 1988a). In this case, considering that *V*_max_ values during knee extension have been reported to be as high as 750°⋅s^-1^ in humans (Hauraix et al., 2017), angular velocity values varied by only 2.6% of *V*_max_ over a relatively wide range of torque values (Seger and Thorstensson, 2000), which is similar to the 1.8% reported by Edman in isolated single muscle fibers (Edman, 1988a). Evidence of a double-hyperbolic F-V relationship during single-joint muscle actions can also be inferred from other studies (Dudley et al., 1990; Yeadon et al., 2006; Forrester et al., 2011).

By contrast, the *in vivo* human F-V relationship during multi-joint muscle actions has been reported to follow a strictly linear pattern (Bobbert, 2012). However, recent findings suggest that the observed linearity may result from failure to obtain experimental data in the extreme (high-force/low-velocity and low-force/high-velocity) regions of the F-V curve. Indeed, Hahn et al. (2014) demonstrated that when a low enough force or high enough velocity is evaluated, the F-V relation becomes hyperbolic in the range of moderate to low forces. With regard to the high-force/low-velocity region, a recent study measured the forces realized during multi-joint exercise performed over a small section of the ROM covering the optimal angle (Alcazar et al., 2018b). This setup enabled the recording of peak force values as high as 89–96% of *P*_0_ at corresponding velocities as low as 1–4% of *V*_max_. The hyperbolic F-V equation was observed to overestimate isometric force values by 13%, with measured F-V data deviating below the rectangular hyperbola at forces above 90% of *P*_0_ and velocities below 5% of *V*_max_ (Alcazar et al., 2018b). Collectively, these findings suggest that apparently linear F-V relationships observed during multi-joint exercises may be a misconception resulting from the relatively narrow range of concentric forces that is usually evaluated (∼40–90% of *P*_0_). In this sense, while a linear fitting might be an excellent representation of the F-V relationship at forces above ∼40% of *P*_0_ due to the deviation of F-V values below the rectangular hyperbola above 90% of *P*_0_, the F-V values will deviate progressively from the linear model below ∼40% of *P*_0_ as they approach *V*_max_. In contrast with the latter, a recent study concluded that the F-V relationship during a multi-joint exercise (bench press) was linear even when a set of loads ranging from 12 to 83% of *P*_0_ was considered (Cuevas-Aburto et al., 2018). The authors based their conclusion on the observation of individual *R*^2^ values ranging from 0.76 to 1.00, while no comparison was conducted between linear and hyperbolic models. By a careful analysis of the data from one subject presented in Figure 1 of that study Cuevas-Aburto et al. (2018) we observed that the fit of a hyperbolic model was slightly superior to a linear model (*R*^2^ = 1.000 vs. 0.997; standard error of the estimate = 0.012 vs. 0.045), although they also might be considered to be similar. However, the linear model underestimated *P*_0_ (-7%) and *V*_0_ (-5%) and overestimated *W*_max_ (+4%), optimal force (+6%) and optimal velocity (+12%) compared with the hyperbolic model. These discrepancies are likely to be much higher in those individuals showing inferior *R*^2^ values from the linear model (i.e., *R*^2^ = 0.76–0.95). Therefore, although linear models might be adequate in some individuals because of their feasibility and similar output results compared with hyperbolic models, the F-V relationship is in fact curvilinear in the range of moderate-to-low forces.

Another factor that may influence the shape of F-V relation during *in vivo* measurements is the joint angle at which F-V data are obtained. Firstly, joint angles should not be inferred from dynamometer crank angles during isokinetic testing due to potential differences between both measures (up to 20°) (Pain et al., 2013). Secondly, it is important to note that due to the influence of the in series elastic component of the muscle-tendon complex (Reeves and Narici, 2003) it is not possible to infer identical muscle length from equivalent joint angles when measurements are recorded at different contraction velocities. Tendons are visco-elastic structures that exhibit both rate-dependent (viscous) and rate-independent (elastic) properties. Several ultrasound studies (Hauraix et al., 2013, 2015, 2017; Fontana Hde et al., 2014) noted that tendons undergo lengthening during the early muscle force development until muscle force reaches its peak. When muscle force decays tendons shorten releasing the elastic energy previously stored (Finni et al., 2003; Earp et al., 2017). This mechanism of tendon recoil enables muscle fascicles to shorten at lower velocities at given muscle-tendon unit velocities, which enhances force production. Muscle-tendon interaction also implies that muscle length at a given joint angle is shorter under higher forces (i.e., slower velocities) because of the greater tendon lengthening. Other structures within muscles exhibiting spring-like properties may amplify this effect (e.g., actomyosin cross-bridges, actin and myosin filaments, titin, and the connective tissue scaffolding of the extracellular matrix) (Roberts, 2016). Fortunately, ultrasound studies have shown that peak torques during concentric knee extensions and plantar flexions at different angular velocities occurred when vastus lateralis and medial gastrocnemius fascicle lengths, respectively, were close to their optimal fascicle lengths (Ichinose et al., 2000; Fontana Hde et al., 2014). Thus, collecting F-V data at the point of peak torque may allow for the effects of velocity to be studied in isolation. In any case, the F-V relationship obtained from peak values or angle-specific values has been reported to display essentially the same shape (with minor differences in curvature), and differ only in magnitude (Wyatt and Edwards, 1981; Yates and Kamon, 1983; Westing et al., 1988; Westing and Seger, 1989; Hortobagyi and Katch, 1990; Webber and Kriellaars, 1997).

With the advent of modern imaging techniques, increased efforts have been made to study the F-V relationship *in vivo* through the combined use of ultrasound and dynamometry (Ichinose et al., 2000; Finni et al., 2003; Fontana Hde et al., 2014; Hauraix et al., 2015, 2017). However, the estimation of fascicle force from external joint torque relies on several important assumptions. First, moment arms and the relative contribution of the target muscle to external force must be assumed to be constant across subjects, although recent research points to substantial inter-individual differences in these parameters (Massey et al., 2015; Trezise et al., 2016). Even within subjects, the relative contribution of individual agonist muscles or muscle fascicles to external force production at different contraction velocities might not be the same, because their F-V properties may differ due to distinct characteristics of muscle architecture and ATPase activity (Barany, 1967; Spector et al., 1980). The activity of antagonist muscles lowering joint torque is usually not considered. Moreover, muscle architecture and fascicle behavior along the muscle are presumed to be uniform, in spite of reports demonstrating great intramuscular heterogeneity (Trezise et al., 2016; Moo et al., 2017). These differences can even be magnified by the arrangement of muscles around the joints (Lieber and Friden, 2000). In addition, it should be noted that force produced by a fascicle cannot be directly inferred from the degree of its shortening: if the muscle fibers under investigation were shortening at their maximal unloaded velocity they would not transmit forces to their myotendinous junctions. Indeed, studies in prepared frog muscles suggest that single fibers might even be shortening at velocities much greater than their unloaded contraction velocities, due to assisting force provided by the fastest fibers (Edman, 2014). In the light of these methodological challenges, the validity of muscle fascicle F-V relationships as estimated from ultrasound measurements must be doubted. The inability of ultrasound measurements to capture out-of-plane movements of muscle fascicles or the lack of a fixed frame of reference should also be considered (Karamanidis et al., 2005).

On the other hand, the effect of different muscle lengths on the double-hyperbolic F-V relationship has not been thoroughly studied yet, although Hahn et al. (2014) found that the curvature of the F-V relationship varied across different knee joint angles. This may be due to the history dependence of muscle contraction (Rassier and Herzog, 2004), by which force depression is observed after active muscle shortening (Edman et al., 1993; Herzog and Leonard, 1997; De Ruiter et al., 1998). This effect is more pronounced when greater mechanical work is performed (Herzog et al., 2000) and may lead to a higher curvature when the F-V data are obtained at shorter muscle lengths after active muscle shortening (Racz et al., 2002; Hahn et al., 2014).

## The Eccentric F-V Relationship

The eccentric portion of the F-V relationship has not been studied as extensively as the concentric part. However, eccentric muscle function is vital during various activities of daily living such as absorbing energy when landing from a jump or lowering an object or body mass, or for proper antagonist muscle function. Early studies conducted in animal muscles showed that the eccentric portion of the F-V relation follows a convex upward curve with force values rising substantially above isometric levels in the range of low negative contraction velocities, while force values remain practically unchanged in the range of moderate-to-high negative contraction velocities (Levin and Wyman, 1927; Katz, 1939). Edman (1988a) described the transition from concentric to eccentric forces as a sigmoidal function with inflection at *P*_0_, and noted that force values increased steeply at low negative velocities (up to 1.2 *P*_0_), but smoothly at moderate-to-high negative velocities (up to 1.6–1.8 *P*_0_) (Figure 6B). These findings were confirmed by other studies (Lannergren, 1978; Stienen et al., 1992; Curtin and Edman, 1994; Krylow and Sandercock, 1997; Rijkelijkhuizen et al., 2003). A specific, albeit rarely used, hyperbolic equation has been proposed to describe the eccentric F-V relationship in the range of forces between 1.0 and 1.6 *P*_0_ (Mashima et al., 1972):

(3) $$V=\frac{b^{\prime }(P_{0}-P)}{2P_{0}-P+a^{\prime }}$$

where *a′* and *b′* are specific constants of the eccentric F-V relationship with the dimensions of force and velocity, respectively. Thus, the ratio *a′*/*P_0_* indicates the curvature of the eccentric F-V relationship. Other hyperbolic equations for modeling the eccentric F-V relationship with an asymptote set at 1.5 *P*_0_ have been reported (Cole et al., 1996).

To our knowledge, the first study evaluating the eccentric portion of the F-V relationship in humans was that conducted by Komi (1973) using an isokinetic dynamometer that measured force during eccentric and concentric elbow flexions. The results provided by Komi showed eccentric force values approximately 20–30% greater than the estimated maximal isometric force, with force rising more steeply at lower compared with higher negative velocities. This enhanced eccentric force response was accompanied by similar EMG values being recorded in agonist and antagonist muscles at the different concentric and eccentric velocities (Komi, 1973). Similar results were reported by Rodgers and Berger (1974) soon after the study by Komi. In women, by contrast, maximal eccentric force was only 10% greater than maximal isometric force (Griffin, 1987).

Studying both men (Westing et al., 1988) and women (Westing and Seger, 1989) performing knee extensions, Westing and colleagues found eccentric torque values to be consistently greater by 4–18% than isometric ones, although neither differences in peak nor angle-specific torque values reached statistical significance. Apparently greater maximal eccentric torque values compared with the isometric torque were also reported during elbow flexion and extension (16–24%) (Hortobagyi and Katch, 1990), forearm supination (8–25%) (Thomson and Chapman, 1988), ankle dorsiflexion (27%) (Reeves and Narici, 2003), knee extension (14–20%) (Holder-Powell and Rutherford, 1999; Finni et al., 2003), and multi-joint leg extension (26–30%) (Hahn et al., 2010); as compared to this large body of evidence, only one study reported knee extension moments to be 5% lower in maximal eccentric as compared to maximal isometric contractions (Webber and Kriellaars, 1997). The observation that in studies performed in humans maximal eccentric force values were consistently lower than those reported in animal studies (∼1.3 vs. ∼1.6–1.8 *P*_0_) led several researchers to propose a central inhibitory mechanism impairing *in vivo* eccentric force production (Perrine and Edgerton, 1978; Wickiewicz et al., 1984; Westing et al., 1988; Westing and Seger, 1989; Hortobagyi and Katch, 1990; Yamauchi et al., 2007).

To elucidate such mechanisms, various investigations have studied the F-V relationship using electrical muscle stimulation applied either in isolation or superimposed on voluntary eccentric contractions. These studies confirmed that torques produced during electrically induced/assisted contractions were significantly greater than those achieved in maximal voluntary contractions (Dudley et al., 1990; Westing et al., 1990; Seger and Thorstensson, 2000; Pain et al., 2013). In addition, some studies found EMG values to be 10–30% lower during voluntary eccentric compared with velocity-matched concentric muscle actions (Westing et al., 1991), although this was not the case in other studies (Reeves and Narici, 2003; Chino et al., 2008). Lending further support to the neural inhibition hypothesis, both the eccentric F-V relationship obtained from electrically induced/assisted contractions and the EMG-corrected eccentric F-V relationship obtained from voluntary contractions were found to be more similar to that observed in animal studies (Dudley et al., 1990; Pain and Forrester, 2009; Pain et al., 2013). Discrepant torque augmentation through electrical stimulation observed in elite athletes and sedentary subjects (Amiridis et al., 1996) further suggests that neural inhibition of eccentric contractions may depend on the training status. Confirmatively, neuromuscular activation (Aagaard et al., 2000) and force production in eccentric contractions (Spurway et al., 2000) were improved after resistance training. Recently, Hahn (2018) presented data on the effect of test familiarization on eccentric muscle strength, which showed that torques were enhanced up to 20% after four familiarization sessions, with some subjects reaching eccentric torque values 40% greater than the isometric torque. In addition to learning effects, the preceding state (i.e., at rest vs. submaximally/maximally activated) of muscle may influence eccentric performance (Hahn, 2018). Indeed, when muscle actions started from a resting state or a low preload, the muscle first shortened presumably stretching the tendon, even if joint rotation indicated the onset of the eccentric contraction (Hahn, 2018). In such contractions, eccentric forces may very well be similar or even lower than the maximal isometric force, since they actually correspond to a concentric muscle action. Therefore, a maximal activation state (≥95% of the isometric force) preceding each eccentric contraction is recommended for proper testing (Hahn, 2018). Another methodological consideration to keep in mind is that, while registering angle-specific force values during eccentric contractions may help to acquire data at similar muscle length, this may differ from the muscle length recorded at the same joint angle during isometric and concentric contractions (Reeves and Narici, 2003).

Another explanation that may contribute to the lower forces observed in *in vivo* as compared to *in vitro* studies is related to temperature effects. Studies on the eccentric portion of the F-V relationship in different isolated muscle preparations are usually conducted at temperatures below 25°C (Lannergren, 1978; Edman, 1988a; Stienen et al., 1992; Curtin and Edman, 1994). In this sense, De Ruiter and De Haan (2000, 2001) and Ruiter et al. (2000) found maximum eccentric force values to be ∼40% higher than maximal isometric ones when the human adductor pollicis muscles was tetanically activated at physiological temperatures, while they were 62% higher than the isometric force when muscle temperature was lowered to 22.3°C. Thus, Cook and McDonagh (1995) found eccentric force values 80% greater than the isometric force in tetanically activated human first dorsal interosseous muscles at 27.6°C. In addition, eccentric torque enhancement may also be related to the amplitude of muscle stretch. A positive relationship between stretch amplitude and eccentric torque enhancement has been demonstrated for electrically evoked (Cook and McDonagh, 1995) and maximal voluntary muscle actions (Hahn et al., 2014). Finally, maximal eccentric forces appear to level off or decrease beyond a certain level of lengthening velocity (Dudley et al., 1990; Holder-Powell and Rutherford, 1999; Hahn et al., 2014), suggesting a velocity-dependence of eccentric torque enhancement. In this regard, reduced cortical and spinal excitability during eccentric contractions (Duchateau and Enoka, 2016) may still be present in novice subjects and/or above a certain threshold of muscle lengthening velocity.

## Molecular Insights Into the F-V Relationship

The sliding filament theory is a physico-chemical theory accounting for the mechanical, chemical, and structural features of skeletal muscle that was formulated by Huxley (1957). The theory was inspired by previous evidence showing the microscopic structure of skeletal muscle and chemical reactions observed in glycerinated muscle preparations (Szent-Gyorgyi, 2004), and the relationship observed between force, velocity, and heat production (Hill, 1938). According to Huxley’s theory, muscle contraction is due to cross-bridges being formed between actin and myosin filaments following muscle excitation and energy availability. This leads to the movement of actin relative to myosin filaments, until the link is broken due to a chemical reaction (Huxley, 1957). Huxley postulated that “the total tension in the muscle will be the sum of the tension generated by all the contraction sites within one half-sarcomere” (Huxley, 1957). According to the theory, the decrease in force observed at increasing contraction velocity is caused by: (1) the increasing likeliness of pairs of actin and myosin myofilaments passing each other without cross-bridges being formed; and (2) the increasing proportion of links formed between actin and myosin that will not be disassociated in time, generating a force in the opposite sense of muscle shortening. Then, the maximal velocity of unloaded shortening is found at the point where negative forces equal positive forces, and net force is zero (Huxley, 1957). The agreement between the experimental F-V data reported by Hill (1938) and the sliding filament theory (Huxley, 1957) was decisive for the acceptance of the theory. For that purpose, the rate constants *f* (rate constant for the formation of cross-bridges) and *g* (rate constant for the detachment of cross-bridges) were given specific values that varied depending on the distance between the active site on the actin filament and the equilibrium position of the sliding element on the myosin filament (Huxley, 1957; Gordon et al., 1966; Podolsky et al., 1969; Huxley and Simmons, 1971). Thus, it is currently accepted that muscle contraction results from the relative sliding of two sets of filaments arranged in parallel in each sarcomere: the thick filament (in skeletal muscles mainly composed of the motor protein myosin II) and the thin filament (containing actin filaments). Upon activation, myosin heads repeatedly attach to actin, stroke and then detach again, determining muscle performance (i.e., force), which decreases with increasing shortening velocities in a hyperbolic manner.

**FIGURE 7:**
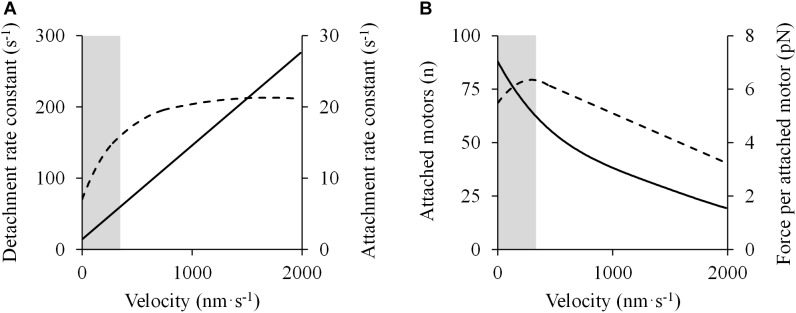
Molecular mechanisms accounting for the double-hyperbolic shape of the F-V relationship. Data were obtained from Figure 4 in Piazzesi et al. (2007) using specialized software (ImageJ 1.51q8, NIH, United States). In this modified version: **(A)** Cross-bridge kinetics (detachment – *solid line* – and attachment – *dashed line* – rate constants) are presented as a function of velocity. **(B)** The number of attached motors (*solid line*) varies with velocity, following a nearly hyperbolic shape, while the average force per attached motor (*discontinuous line*) decreases below a certain velocity threshold. These events explain the deviation from the rectangular hyperbola in the high-force/low-velocity region of the force-velocity relationship (*shaded area*).

However, as shown previously, deviations from the rectangular hyperbola in different portions of the F-V relation have been found. These deviations may be due to the violation of one or more of three central assumptions regarding the kinetics of cross-bridge formation (Seow, 2013): (1) the detachment rate is linearly proportional to the shortening velocity; (2) the attachment rate is independent of shortening velocity; and (3) force per cross-bridge declines linearly with shortening velocity. In fact, recent evidence has shown that these assumptions may be incorrect in the high-force/low-velocity portion of the F-V relationship. Piazzesi et al. (2007) conducted a series of experiments with X-ray interference and mechanical measurements of intact single muscle cells to evaluate the molecular basis of the F-V relationship in skeletal muscle. They found that the detachment rate decreased linearly with decreasing shortening velocity, but never dropped to zero (Figure 7A) (Piazzesi et al., 2007), which contrasts with the original model in which the detachment rate was zero at *P*_0_ (Huxley, 1957). In addition, the attachment rate of myosin motors increased with shortening velocity until ∼0.5 *P*_0_, below which it stabilized and remained constant at faster shortening velocities (Figure 7A) (Piazzesi et al., 2007), whereas the original model assumed that the attachment rate was constant across various shortening velocities from zero load to *P*_0_ (Huxley, 1957). Finally, the force exerted per cross-bridge (expected to decrease with shortening velocity) was found to be nearly constant at intermediate and low velocities (Figure 7B) (Piazzesi et al., 2007). These observations gave support to the double-hyperbolic F-V relationship.

To test whether the double-hyperbolic F-V relation observed in skeletal muscles and single muscle fibers (Edman, 1988a; Devrome and MacIntosh, 2007) was also evident at the single sarcomere level, an *in vitro* motility assay system was developed to investigate the steady-state F-V relation derived from the interaction between actin and myosin molecules (Chaen et al., 1989; Oiwa et al., 1990; Ishii et al., 1997). F-V data resembled a hyperbola at forces lower than 0.8 *P*_0_, but fell below the hyperbola at forces greater than 0.8 *P*_0_ (Ishii et al., 1997). The F-V relationship was found to be analogous in shape to the double-hyperbolic F-V relationship observed in single muscle fibers (Edman, 1988a). Because of the small number (∼4–10) of myosin molecules involved in the bead movement, the authors suggested that the double-hyperbolic F-V relation might be intrinsic to the kinetic properties of the individual cross-bridges (Ishii et al., 1997). Conflicting with this hypothesis, other studies have provided evidence to show that the shape of the F-V relation is rather influenced by the change in the number of attached cross-bridges and the force created by each of them. Edman (1993) conducted a series of experiments in which fiber force and stiffness were recorded while fibers shortened at various velocities during tetanic contraction. Since stiffness indicates the proportion of active cross-bridges within the fiber (Ford et al., 1981), the resultant plot of stiffness against force allowed for conclusions about the nature of sarcomeric force production: the proportion of attached cross-bridges increased with increasing force but beyond a certain breakpoint (corresponding to 0.8 *P*_0_) the effect of greater cross-bridge attachment was attenuated by a decreasing force per cross-bridge formed, which would explain the double-hyperbolic F-V relation observed in skeletal muscles (Edman, 1993). These findings were later confirmed in both rested and moderately fatigued intact single fibers (Curtin and Edman, 1994). Piazzesi et al. (2007) found a 40% increase in the number of cross-bridges formed between 0.8 and 1.0 *P*_0_, accompanied by a 12% decrease in the force produced by each myosin stroke (Figure 7B) (Piazzesi et al., 2007). This modulation may explain the bend of the F-V relationship at high forces and its appearance as a double-hyperbolic curve. In line with this, Mansson (2010) presented a four-state cross-bridge model that considered both the velocity-dependent attachment rate and the variation of the proportion of cross-bridges attached to the actin filaments at different force-generating states. The proportion of cross-bridges in a low force-generating state decreased, while that of cross-bridges in a high force-generating state increased between 0.85 and 1.0 *P*_0_ (Mansson, 2010), which led to a net increase in the number of cross-bridges concomitant with a decrease in the average force exerted per cross-bridge.

On the other hand, enhanced force production during eccentric contractions may be due to an increased number of cross-bridges attached to actin, increased force per cross-bridge, or a combination of these (Rassier, 2017). Several studies have reported an increased number of myosin heads attached to actin during eccentric compared with isometric contractions (Linari et al., 2000; Brunello et al., 2007). This would be facilitated by the attachment of the second motor domain of the myosin, which remains inactive under isometric and concentric conditions, but is activated with stretch. It is interesting to note that, despite the increased number of attached cross-bridges, ATP consumption is actually lower during eccentric contractions compared with isometric contractions (Linari et al., 2003). This is because myosin heads detach from actin by a strain-dependent process that does not require ATP splitting (Piazzesi et al., 1992), and thus ATP use during active muscle lengthening is attributed to the activity of the Ca^2+^ pump (Linari et al., 2003). In support of the theory on multiple force-generating states of cross-bridges, myosin heads that are only weakly bound to actin (pre-power stroke state) under isometric conditions have been reported to switch to a strong binding state with stretch (Getz et al., 1998; Linari et al., 2004; Rassier, 2008), leading to an increased force per cross-bridge during lengthening contractions. Notably, having a larger fraction of myosin heads in a weak binding state under isometric conditions has been related to a greater force production during active muscle lengthening, which depended on the myosin heavy chain isoform (Linari et al., 2004). Regardless the cross-bridge-related mechanism, force enhancement in lengthening contractions has been described to occur in two phases (Getz et al., 1998; De Ruiter and De Haan, 2000, 2001; Pinniger et al., 2006): firstly, there is a steep and substantial increase in force until a critical amount of stretch is reached (phase I), beyond which cross-bridges detach mechanically from actin (phase II) and either a smooth and modest increase in force or no change can be noted. Consequently, detached cross-bridges must reattach rapidly to actin to keep producing force. Fortunately, reattachment during muscle lengthening occurs at a higher rate compared with reattachment under isometric or concentric conditions (Lombardi and Piazzesi, 1990). Force exerted at the transient point between phases I and II increases rapidly with increasing velocity (detachment rate-dependent) until it levels off beyond a lengthening velocity of ∼1.6–2.0 *P*_0_. By contrast, force exerted during the second phase as a consequence of cross-bridge reattachment decreases as a function of lengthening velocity (attachment rate-dependent).

The mechanism explaining the deviation of the F-V curve from the rectangular hyperbola in the low-force/high velocity region is less clear since no experimental data concerning the rate constants at very high velocities (i.e., <0.05 *P*_0_) exist. According to Huxley’s theory, maximal unloaded shortening velocity is attained when the resulting force from the attached cross-bridges pulling in the right sense and those negatively strained – pulling in the opposite sense – is zero (Huxley, 1957). Thus, the higher the ability of the myosin heads to rapidly detach from actin, the higher the maximal unloaded shortening velocity. This ability is controlled by the hydrolysis of ATP (with the release of Pi and ADP) and the new binding of ATP, which is predominantly influenced by the activity of myosin ATPase (Barany, 1967; Schiaffino and Reggiani, 1996) and favored by the negative strain of the myosin lever arm (i.e., myosin mechanosensing) (Caremani et al., 2015). However, contrary to the expectation of high metabolic costs that would result from this mechanism, the rate of ATP splitting is low during rapid or unloaded muscle shortening (Kushmerick et al., 1969; Rall et al., 1976; Homsher et al., 1981). It has recently become clear that the thick filaments have a second mechanosensing mechanism that is distinct from that of the individual myosin heads, and that is also independent from the thin filament-based calcium-dependent regulatory mechanism of muscle contraction (Irving, 2017). Thick filaments remain in a structural and functional OFF state at rest, with the two heads of the myosin locked in a conformation in which they can neither bind to actin nor hydrolyze ATP even in the presence of high intracellular calcium concentration (Irving, 2017). Thick filaments are progressively switched ON (i.e., myosin heads become available to cross-bridge formation) with increasing mechanical stress (Linari et al., 2015; Fusi et al., 2016). This thick filament-based muscle contraction mechanism would be especially important to control the metabolic cost of muscle contraction. Thus, the number of myosin heads attached during *V*_0_ has been found to be as low as ∼1–6% of those attached during *P*_0_ (i.e., ∼1–4 motors), thereby reducing the metabolic cost of muscle contraction, which is achieved by switching OFF the majority of myosin heads despite high intracellular calcium concentration (Fusi et al., 2017). The exact mechanism of this thick filament mechanosensing is poorly understood, since scant data of the molecular structure of the thick filament exist. Other proteins such as titin and myosin binding protein-C have been suggested as potential regulators of mechanosensitivity (Irving, 2017).

The mechanosensing of the thick filament is likely to be associated with the deviation of F-V data from the rectangular hyperbola at forces below 0.05 *P*_0_ and the differences between *V*_max_ and *V*_0_ values (Edman, 1979; Claflin and Faulkner, 1985). To better understand this relationship, it is important to be aware of motor unit recruitment patterns during voluntary muscle actions. During ramp contractions, motor units are recruited in dependency of force demands based on the size of the motoneuron soma (Henneman’s or size principle) (Henneman, 1957; Henneman et al., 1965). Thus, motor units are orderly recruited (i.e., from small to large motor units) at a certain and reproducible force threshold while motor unit firing rates progressively increase as the muscle produces more force (Desmedt and Godaux, 1977, 1978). However, in situations where rapid force development is required (i.e., ballistic condition), the recruitment pattern may vary (Hodson-Tole and Wakeling, 2009). Ballistic muscle actions are characterized by initially high firing frequencies (Oishi et al., 1988) decreasing during contraction (Desmedt and Godaux, 1977), and a substantial decrease of recruitment thresholds of larger motor units compared with ramp contractions (Desmedt and Godaux, 1977, 1978; Yoneda et al., 1986; Harwood and Rice, 2012). This event allows an earlier recruitment of most motor units even before force production can be detected, with some reversals in the order of recruitment between smaller and larger motor units (Desmedt and Godaux, 1977; Yoneda et al., 1986). The early activity of large motor units does not necessarily conflict with Henneman’s principle. Since larger motor units possess faster conduction velocities, they may produce force earlier than smaller motor units even if recruited at a slightly later time (Desmedt and Godaux, 1979). The compression of the range of motor unit recruitment thresholds and the early activation of faster motor units would be an advantage during very fast ballistic muscle actions that are so brief for the motor unit recruitment pattern to be amenable to modification through proprioceptive feedback. In this context, the thick filament-based calcium-independent regulatory mechanism of muscle contraction (Irving, 2017) has a great relevance to control the recruitment of active single muscle fibers, and at the same time to reduce the metabolic cost of muscle contraction at low forces/high velocities. Under low loads or unloaded conditions (i.e., in fast to very fast muscle actions), the mechanical load would be early sustained by the faster muscle fibers, whose thick filaments would be switched ON to make their myosin heads available for actin binding and force production. By contrast, the slower muscle fibers would not perceive any mechanical stress, and thus their thick filaments would remain in an OFF state. Therefore, this mechanism would allow the fastest muscle fibers to contract under very low loads or unloaded conditions with no or little resistive forces coming from the slower muscle fibers. This hypothesis would explain both the discrepancies found in the literature between *V*_max_ and *V*_0_ values (Edman, 1979; Claflin and Faulkner, 1985), and the ability of single muscle fibers to shorten above their maximal unloaded shortening velocity without being damaged (Edman, 2014).

One further factor that might influence the shape of the F-V relationship is the history-dependent behavior of muscle contraction (Rassier and Herzog, 2004): isometric force at a given muscle length is lower when the contraction is preceded by muscle shortening (force depression after active muscle shortening) (Abbott and Aubert, 1952); while isometric force at a given muscle length is higher when the contraction is preceded by muscle lengthening (residual force enhancement after active muscle lengthening) (Edman et al., 1982). These aspects are rarely considered in cross-bridge models of muscle contraction. To explain the force depression, several hypotheses have been proposed that are related to the non-uniformity of sarcomere length, the accumulation of metabolites or the stress-induced inhibition of cross-bridge attachment, with the latter hypothesis being most supported by scientific evidence (Rassier and Herzog, 2004). The diminution of attached cross-bridges (Sugi and Tsuchiya, 1988; Lee and Herzog, 2003) is expected to be caused by the stress that they impose on the portion of myofilaments that is initially not yet inside the overlap zone, but that will reach that zone with the advancement of muscle shortening (Marechal and Plaghki, 1979). In addition, the PEVK region of titin might attach to actin filaments, thus inhibiting cross-bridge formation and leading to force depression (Rode et al., 2009). Hence, higher forces (i.e., higher stresses) and greater amounts of shortening (leading to a greater proportion of inhibited cross-bridges entering the overlap zone) are associated with higher force depression after active shortening (Herzog et al., 2000). On the other hand, sarcomere length non-uniformity and instability, an increase in the proportion of attached cross-bridges, or an engagement of a passive element have been proposed as possible contributors to residual force enhancement after active muscle lengthening (Rassier and Herzog, 2004). Indeed, the combination of an active component that involves the previously reported increase in the proportion of attached cross-bridges, together with a passive component likely related with a Ca^2+^-induced increase in the stiffness of the protein titin (Hessel et al., 2017), is suggested as the main mechanism involved in residual force enhancement (Rassier and Herzog, 2004; Hessel et al., 2017).

## Final Considerations

Due to the breadth of the topic, we were forced to omit several studies on the F-V relationship that we considered less relevant for the overall topic of this review. We apologize to all authors whose works we were not able to include. Further reviews focusing on other aspects of the F-V relationship or muscle contraction during shortening and lengthening can be found in the literature, among others: (Gulch, 1994; Gordon et al., 2000; Jones, 2010; Schiaffino and Reggiani, 2011; Mansson et al., 2015; Hessel et al., 2017; Hahn, 2018).

Knowledge about the shape of the F-V relationship in skeletal muscles has evolved substantially over the last 100 years. However, the present review reveals that significant discrepancies regarding the shape of the eccentric and concentric F-V relationship still exist in the current literature. The deviations of F-V values from the original hyperbolic F-V relationship at both low and very high positive (concentric) velocities may be due to the fact that the F-V relationship is actually double-hyperbolic (Edman, 1988a) and to differences in the proportion and characteristics of single muscle fibers contributing to force production at different shortening velocities (Josephson and Edman, 1988), respectively. Although good fits of concentric F-V data may be achieved by single-hyperbolic models, in the high-force/low-velocity region experimental data may be better represented by double-hyperbolic models (Edman, 1988a). This bi-phasic relationship has been confirmed in various muscle preparations (Mansson et al., 1989; Ishii et al., 1997; Edman, 2005; Devrome and MacIntosh, 2007), and has recently also been derived using *in vivo* data obtained in humans (Alcazar et al., 2018b). The double-hyperbolic F-V relationship is presumably caused by velocity-specific cross-bridge kinetics (Piazzesi et al., 2007), rather than by a central inhibitory mechanism impairing concentric muscle function, as previously proposed in the literature. A double-hyperbolic F-V relationship is functionally plausible, since it is expected to improve the mechanical stability of the myofilament system at high forces (Edman et al., 1997) by minimizing the redistribution of sarcomere lengths between weaker and stronger segments along the muscle fibers (Edman et al., 1985, 1988; Ahn et al., 2003; Moo et al., 2016, 2017). With regard to the F-V data in the low-force/high-velocity region, the motor unit recruitment during ballistic conditions (Desmedt and Godaux, 1977, 1978) and the thick filament-based muscle contraction mechanism (Irving, 2017) would explain discrepancies found in the literature between *V*_max_ and *V*_0_ values (Edman, 1979; Claflin and Faulkner, 1985) and the low metabolic cost of very fast muscle contractions (Homsher et al., 1981).

The increase in force at negative (eccentric) velocities does not follow the change expected on the basis of a continuation of the concentric F-V relationship, but fits an independent hyperbolic function that levels off at ∼1.6–2.0 *P*_0_ in muscle preparations (Mashima et al., 1972). This makes the muscle an efficient braking system when stretched. For the eccentric part of the F-V relationship, several human studies have shown a reduction, no change or an increase in eccentric force values compared with maximal isometric force. The evidence has demonstrated that these discrepancies may be caused by a lack of familiarization (Hahn, 2018), insufficient muscle conditioning (Amiridis et al., 1996), and differences in testing temperatures (De Ruiter and De Haan, 2000). In any case, maximum eccentric force values should generally be found to be greater than isometric force (up to 1.4 *P*_0_), provided that the assessment has been properly carried out (Hahn, 2018). Neural factors may account for decreases in eccentric force production at high lengthening velocities (Duchateau and Enoka, 2016).

Based on the present literature overview, the following methodological recommendations should be considered to improve standardization of the assessment of F-V data in humans:

(1) Subject familiarization seems to be an important step before acquiring F-V data from voluntary contractions. Although further research is necessary, a minimum of two sessions for the concentric (Alcazar et al., 2018a) and four sessions for the eccentric (Hahn, 2018) F-V relationship may be required to minimize inaccuracies.
(2) Some discrepancies among studies in relation to the shape of the F-V relationship were due to the restrictive range of loads/velocities being evaluated. To date, the range of forces/velocities allowing for accurate representation of the F-V relationship or estimation of *P*_0_ and *V*_0_ values is unclear. Until studies addressing this issue are conducted, we encourage researchers to assess the F-V relation in the highest possible ranges of forces and velocities.
(3) Recording joint angle-specific F-V data may lead to measures being obtained at different muscle length, thus introducing bias, especially during concentric contractions (Reeves and Narici, 2003). The implementation of ultrasonography for controlling muscle length or the collection of F-V data at the point of peak force may help minimize possible interactions between the F-V and the force-muscle length relationships (Ichinose et al., 2000; Fontana Hde et al., 2014). The registration of mean F-V data from each performed dynamic muscle action may also provide relevant information on the subjects’ neuromuscular performance over the whole ROM.
(4) A maximal activation state (≥95% of the isometric force) preceding each eccentric contraction is recommended (Hahn, 2018), to ensure that force/torque values are actually recorded during muscle lengthening.

## Author Contributions

JA and RC wrote the manuscript based on relevant investigations for the overall topic of this work. IA and LA supervised and made substantial contributions to the final version of the manuscript. All authors listed approved the manuscript for publication.

## Conflict of Interest Statement

The authors declare that the research was conducted in the absence of any commercial or financial relationships that could be construed as a potential conflict of interest.

## Footnotes

1 An auxotonic contraction refers to a muscle contraction against a varying load.

2 Cineplastic amputation is a surgical technique in which muscles and tendons are arranged in a stump in such manner that it is possible to impart motion and direction to an artificial limb.
